# Urban green valuation integrating biophysical and qualitative aspects

**DOI:** 10.1080/22797254.2017.1409083

**Published:** 2017-12-12

**Authors:** Stefan Lang

**Affiliations:** ^a^ Department of Geoinformatics – Z_GIS, University of Salzburg, Salzburg, Austria

**Keywords:** Green perception, telic representation, geons, regionalization, VHR satellite data, geonalytics

## Abstract

Urban green mapping has become an operational task in city planning, urban land management, and quality of life assessments. As a multi-dimensional, integrative concept, urban green comprising several ecological, socio-economic, and policy-related aspects. In this paper, the author advances the representation of urban green by deriving scale-adapted, policy-relevant units. These so-called geons represent areas of uniform green valuation under certain size and homogeneity constraints in a spatially explicit representation. The study accompanies a regular monitoring scheme carried out by the urban municipality of the city of Salzburg, Austria, using optical satellite data. It was conducted in two stages, namely SBG_QB (10.2 km², QuickBird data from 2005) and SBG_WV (140 km², WorldView-2 data from 2010), within the functional urban area of Salzburg. The geon delineation was validated by several quantitative measures and spatial analysis techniques, as well as ground documentation, including panorama photographs and visual interpretation. The spatial association pattern was assessed by calculating Global Moran’s I with incremental search distances. The final geonscape, consisting of 1083 units with an average size of 13.5 ha, was analyzed by spatial metrics. Finally, categories were derived for different types of functional geons. Future research paths and improvements to the described strategy are outlined.

## Introduction

### The relevance of EO-based urban green mapping and monitoring

*Urban green* as a metonymic expression connotes a holistic, integrated concept comprising ecological, socio-economic, and policy-related aspects: (1) urban ecosystems and biodiversity, manifested in the physical green, as in the vegetative environment; (2) the psychological well-being and quality of life including restorative effects and *restoration of attention* (Kaplan, 1995) induced by green structures and non-monotonous urban landscapes (Carrus et al., 2015); and (3) green mobility and liveability as well as production and consumption including resource maintenance and efficiency in the sense of the green city. Due to this multi-faceted significance, green policies have been established in European and international strategies for sustainable urban neighborhoods under global change dynamics (Luederitz, Lang, & Von Wehrden, 2013), with cities being likewise the main cause and the main solution driver for these sustainability challenges (Grimm et al., 2008).

Remote sensing, in particular satellite-based Earth observation (EO), is a key enabler to encompass the physical dimension of urban green by area-wide mapping and monitoring in multiple nested scales, including the strategic scale(s) of urban planning and management (Nielsen, 2015). Urban green mapping based on EO remote sensing image analysis has become an operational task in city planning, urban land management, and quality of life assessments (Keul & Prinz, 2011). One example of an ambitious European-wide operational urban mapping activity is the Copernicus Urban Atlas information service [see *land.copernicus.eu/local/urban-atlas*]. A comparable land use land cover mapping scheme is applied to so-called functional urban areas (FUAs) with more than 100,000 inhabitants, as well as for most European cities over 50,000 inhabitants. For the city of Salzburg, for instance, it distinguishes the following green-relevant classes: 14,100 (*Green urban areas*) and 14,200 (*Sports and leisure facilities*), 20,000 (*Agricultural, semi-natural and wetland areas*), 30,000 (*Forests*), and 50,000 (*Water*). Urban land cover/use classification recently benefited from representing the third dimension, for example, for green volume mapping, using auxiliary LiDAR data (Hecht, Meinel, & Buchroithner, 2008) or DSM data derived from stereo imagery. Nowadays, VHR optical satellite data in bi- or tri-stereo mode (e.g. Pléiades) allow generating surface models to be used vegetation volume estimation and vertical green structure analysis.

### From green vegetation mapping toward urban green valuation

Urban green features can be mapped in their formal (constitutive) dimension as specific green objects (green elements like trees, patches of grass, etc.) or composite structures (e.g. a group of trees, a lawn between buildings, etc.). Considering their functional and more purpose-related dimension, urban features such as a park, a green belt, green infrastructure, and so on, can be mapped to represent the telic (intentional) dimension (Nielsen, 2015) of city planning. Additionally, specific urban green features may subjectively be conceived differently by citizens with respect to their impression or recreational effects (Keul & Prinz, 2011). In this respect, EO and spatial analysis coupled with qualitative survey-based assessments help address specific aspects of urban green that go beyond functional land use. Lang et al. (2006) show how different EO-derived green types could be further qualified by their semantic meaning in terms of a green impression in the eyes of citizens, by constructing a weighted green index *GI_w_* on a grid-based approach using 50 × 50 m cells. Thereby, a hidden (i.e. *latent*) component was added to a physical representation of urban green structures that is now used to derive units of uniform *green valuation*.

In this paper, the author further advances the representation of the telic dimension of urban green by deriving scale-adapted, policy-relevant units, also known as geons (Lang, Kienberger, Tiede, Hagenlocher, & Pernkopf, 2014). These units shall characterize areas of uniform green valuation under certain size and homogeneity constraints. The research objective is to map urban green valuation, leading to a spatially explicit representation of qualitative aspects of urban green as a latent spatial phenomenon using the geon approach. The operational aim is to achieve a fully exhaustive, yet non a priori, partitioning of the urban space into units that bear internal homogeneity and external difference to neighboring units in terms of their internal green composition. Following the concept of *regions* (Nir, 1987), these units shall serve as homogeneous building blocks for urban planning purposes, adaptive to a scale featuring stability in terms of place-related characteristics, yet small enough to represent the overall urban-scale variability of the concerned phenomenon. The methodological aim is to determine how qualitative assessments obtained from an interview-based survey can be integrated with remote sensing data, so that the biophysical representation and likewise the impression of a specific planning-relevant subset of reality become more obvious in a geon-scape. The paper is structured as follows: After a short reflection on the geographic term region, the geon concept applied in this study is briefly summarized. The core part contains details on how these units of uniform green valuation were delineated, how they can be interpreted, validated and analyzed, and what methodological challenges are connected to this approach.

### Regions – systemic areal units

Boundaries and regions are the flip sides of the same coin. Whether we emphasize boundaries (focus on discontinuity) or regions (focus on continuity) depends on the context and on the algorithmic framework deployed in modeling them. To consider this non-stationary character of geographic phenomena, this paper uses the terms *unit, region*, or *geon* (see later) whenever referring to the homogenous inner part of a whole, and *boundary* when the outer limit is addressed. The nested hierarchy of (socio-geographical) spaces comprises regions and localities as “real entities and objects of active intervention” (Byrne, 1998, p. 93), with the smallest unit being the neighborhood as the lowest significant socio-spatial scale, below which the household level resides as “social atoms” (p. 101). With respect to geographical units, regions can be treated as a group or set of components, coherent by relationships (Nir, 1987). Approaching geographic regions as systems (ibid.) also overcomes the “dichotomy between natural and anthropogenic aspects” (p. 187). We may define boundaries of a region by identifying and delimiting the extension of the interrelations and the processes acting in the region. This contains the distribution of the variables that establish the functioning of the region as a system. In addition, the delineation of regions bears constitutive (composition), telic (function and purpose related), and agentive (process-related) components (Nielsen, 2015). In regional geography, the region is conceptualized as an integral part of a larger whole (Tomanev, 2009), that is “unbound [yet] multiscalar” (p. 149), thus having no pre-given status, at least in the post-structural view. Unbound does not mean arbitrary, though, with the latter minimized by inter-subjective agreement on a conceptual and expert level, as well as numeric parameter optimization to prevent redundancy and other biases.

### Geons as spatial object holons

**Figure 1.**
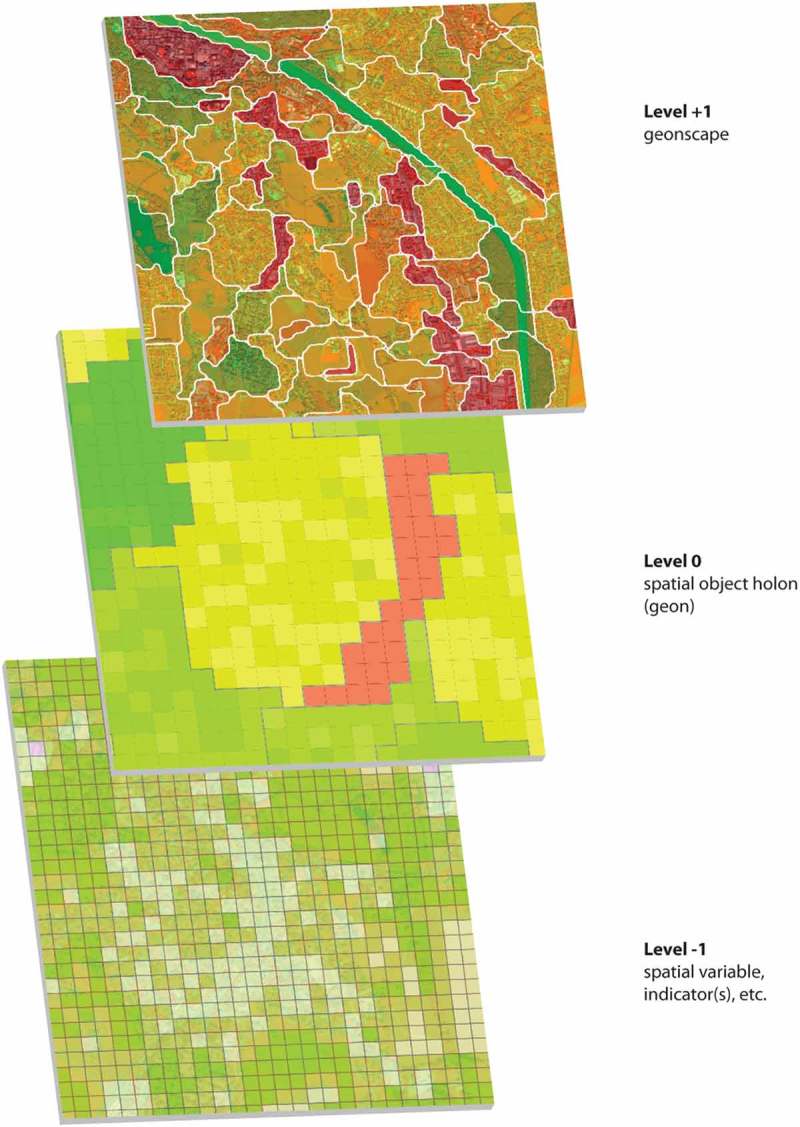
Hierarchical organization of geons. A three-level hierarchy of input layers, geons, and geonscape.

The geon approach (Lang et al., 2014) was developed to spatialize a multi-dimensional indicator set using scale-specific regionalization, in order to visualize (i.e. to *map*) a latent phenomenon in space. An example is societal vulnerability to natural hazards (Kienberger, Lang, & Zeil, 2009). The approach is policy or purpose-driven in unitizing the intervention space, by first decomposing (Weinberg, 1975) a complex phenomenon and then re-composing a meta-indicator based on a scale-specific spatial set of regions. With the geon concept as a holistic regionalization approach, we generate units that are not a priori, but of immanent congruence for policy-related action. Geons show a uniform response regarding the spatial phenomenon under concern and are (ideally) expert validated regarding practical usability and relevance. We may consider regions as *spatial object holons*, assuming that those systemic spatial units bear a dual character *sensu* (Koestler, 1967) in terms of self-assertive (whole-ness) and self-integrative (part-ness) tendencies. Then we apply the three-level concept as proposed in landscape ecology (O’Neill, DeAngelis, Waide, & Allen, 1986) for scaled representations of continuous data. The level of geons, the focal level, is composed of elementary spatial units at the level −1 as the explanatory level (see Figure 1). The constraining level +1, corresponds to the level of the entire geon-scape.

Regions are generated in a spatial context. Here, we deal with two kinds of context: horizontal and vertical, the first referring to spatial embeddedness in a set of neighboring regions, the latter in the sense of hierarchical organization. Hierarchical embeddedness, to be conceived as a function of scale, enables researchers to identify discontinuities that mark the systemic boundaries between conceptual wholes in a (discrete) hierarchy. The scale of interest demarcates a subset of the continuous function, thus sometimes also referred to as the domain of scale (Wiens, 1989). This scale (domain) can be expressed mathematically by applying the concept of a filter, as the “period of time or space over which signals are integrated […] to give message” (Allen & Starr, 1982, p. 18). This conceptualization of scale as an information filter cannot be determined in an absolute sense but depends on the hierarchical organization. Transferred to the geospatial domain, it means that smaller geographical units are constrained by larger ones within a certain hierarchical distance and decreasing spatial granularity (Kuhn & Ballatore, 2015). This spatial granularity goes hand in hand with semantic depth, to be expressed in levels of ontologies.

## Methods

### Study design and materials

**Figure 2.**
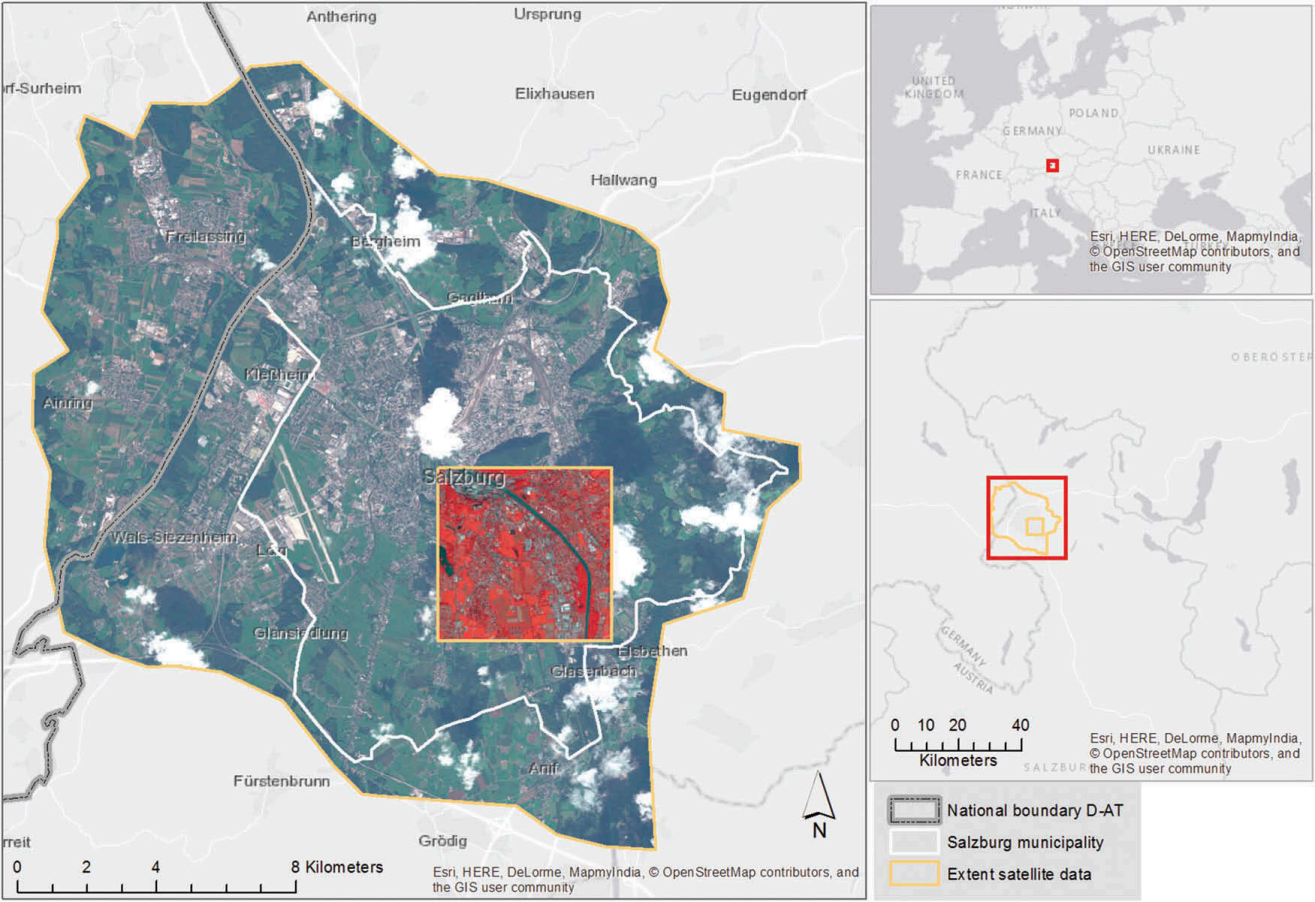
Locations and area of interest (AOI) of the two study areas within the metropolitan area of Salzburg, the test and development AOI SBG_QB, i.e. the small square southeast of the city center using QuickBird data (acquired 21 June 2005 and shown in false-color composite NIR-G-B), and the entire masterplan region AOI SBG_WV with WorldView-2 data (obtained 11 September 2010, displayed in R-G-B).

The study is embedded in monitoring scheme carried out by the urban municipality of the city of Salzburg, Austria, in 5-years intervals since 2005 using VHR optical imagery. Taking into consideration the evolvement of VHR sensors, we have so far used QuickBird, WorldView-2, and Pléiades imagery. The latter serves to enhance green feature extraction capability through elevation information derived from stereo-mode. For the methodological step being discussed in the present paper, we were working on the scene classification layers from 2005, derived from QuickBird data, and 2010, based on WorldView-2. The first one in 2005 was derived for the exact area of the municipality of Salzburg (approx. 65 km^2^) and the second for an extended functional metropolitan area comprising neighboring towns and villages, including the border town of Freilassing on the German side (see Figure 2).

For the first part of the study, SBG_QB, a 10.2 km^2^ sized subset of the entire municipal area was used, defined on pragmatic purposes in a rectangular shape for the development of the method and for validation. The second part, SBG_WV, was conducted on the full extent of the transboundary FUA, the so-called masterplan region Salzburg. This second study area, 140 km^2^ in size, served as a transfer case of the geon delineation to (a) a larger, and functionally defined area, and (b) another data set. The *geonalytics* part of the study was carried out on SBG_WV as well.

**Figure 3.**
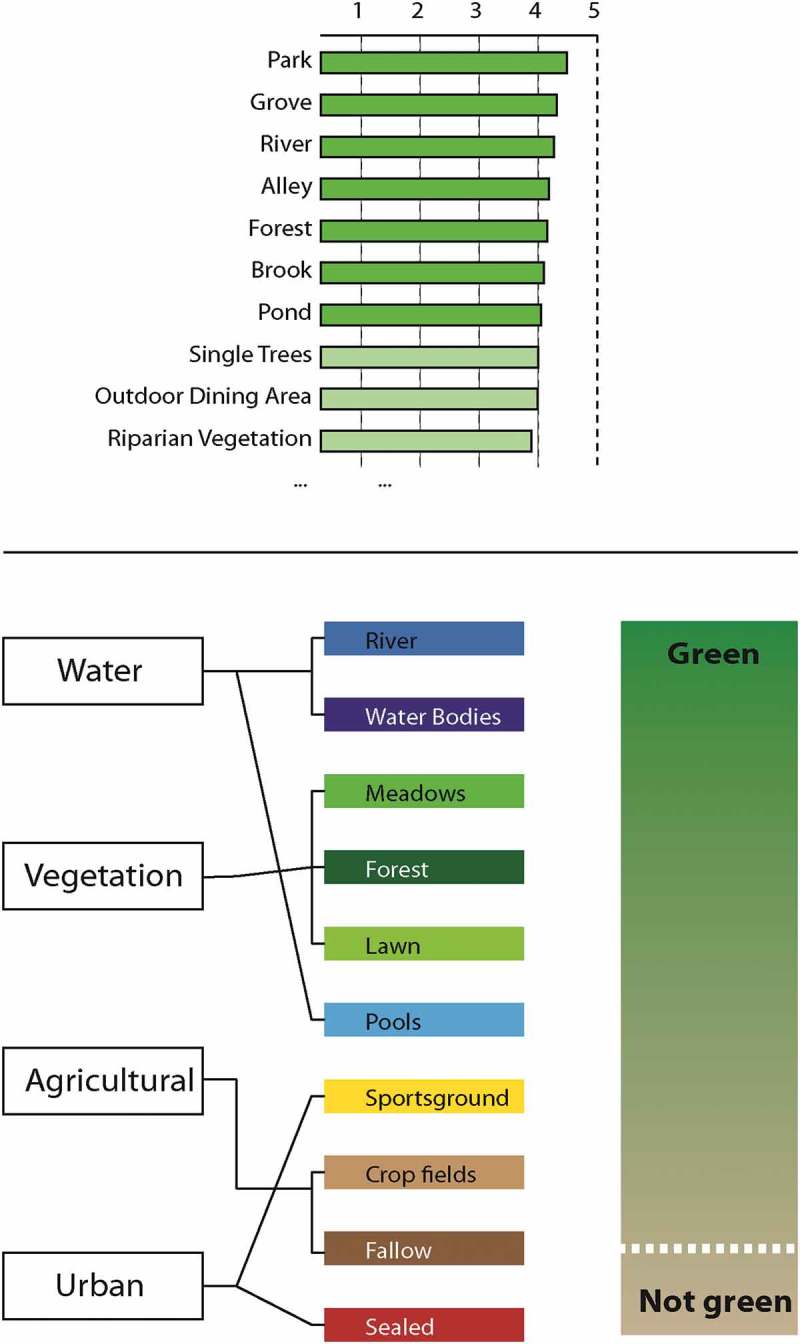
Top: Rating of the green structures (first 10 out of 29) according to their relevance of urban green (Lang et al., 2006; Powell, 2011). Bottom: case-based classification schema and semantic grouping into green and non-green classes.

The results of the classifications using a production system with fuzzy rules (Zadeh, 1965) derived from VHR scenes from 2005 (QuickBird) and 2010 (WorldView-2) were used as input data. The case-based classification scheme was derived from a survey of 128 respondents in 2005 (Lang et al., 2006). In this survey, a total of 29 green structures were assessed by the respondents according to their meaning in terms of green impression or leisure value. The green valuation of these structure types was rated in a range of [1|5]. Based on these 29 structure types, 13 green classes were generated considering spatial and spectral properties, class separability and detectability on remotely sensed imagery (see Figure 3). The classes were semantically grouped into *green* and *non-green* categories according to the meaning assigned to them by respondents from a green impression and leisure value point of view. Advanced arithmetic features were tested based on indices, such as normalized difference vegetation, water, and soil indices (NDVI, NDWI, NDSI), derived from the extended set of spectral bands of WorldView-2. Clouded areas were masked and filled by orthophoto imputation; cloud shadows were treated separately with a modified rule-set, complementary to that of the regular sunlit areas (Powell, 2011). The classification results from both time steps were evaluated by stratified random sampling and point-based accuracy assessment measures (Lang et al., 2007; Powell, 2011). Within the initial study, SBG_QB, we achieved an overall accuracy of 83.2% (Kappa = 0.801). For SBG_WV, within sunlit areas (cloud and cloud shadow areas masked out), the overall accuracy was 92.5% (Kappa = 0.914) with a producer’s accuracy of 92.6% and a user’s accuracy of 92.9%.

### Weighted green index

The empirically gathered green valuation was used to weight the occurrences of green types in defined units, for example, regular grid cells (Lang et al., 2007), but the same can be applied to any other reporting unit, enumeration unit, or city quarters with the known problems when using predefined, administrative units (Hagenlocher, Kienberger, Lang, & Blaschke, 2014). For the calculation of the weighted green index, we used the regular 50 × 50 m reporting grid, which is used by the municipality of Salzburg to map urban planning indicators for various purposes in a spatially distributed manner. Slightly different from the method described in Lang et al. (2006), we calculated the weighted sum of the percentages of occurring green structures per grid cell by multiplying the shares with the respective normalized rating score (see Equation. 1). This applies to all semantically defined green classes including water bodies and rivers. The range is [0|1], whereby zero means no occurrence of a green class within a cell, and 1 means full coverage by the highest rated green class. The value range was then stretched to the upper limit of the original rating, as in [1|5].

(1) $$GI_{w\left(cell\right)}=\overset{n}{\underset{c=1}{\sum }}\left(r_{c}\cdot p_{c}\right)$$

where *GI_w(cell)_* corresponds to the weighted green index per cell, *n* is the number of classes per cell, *r_c_* is the normalized rating, and *p_c_* equals the percentage of class *c* within a cell.

### Regionalization and scaled representation

**Figure 4.**
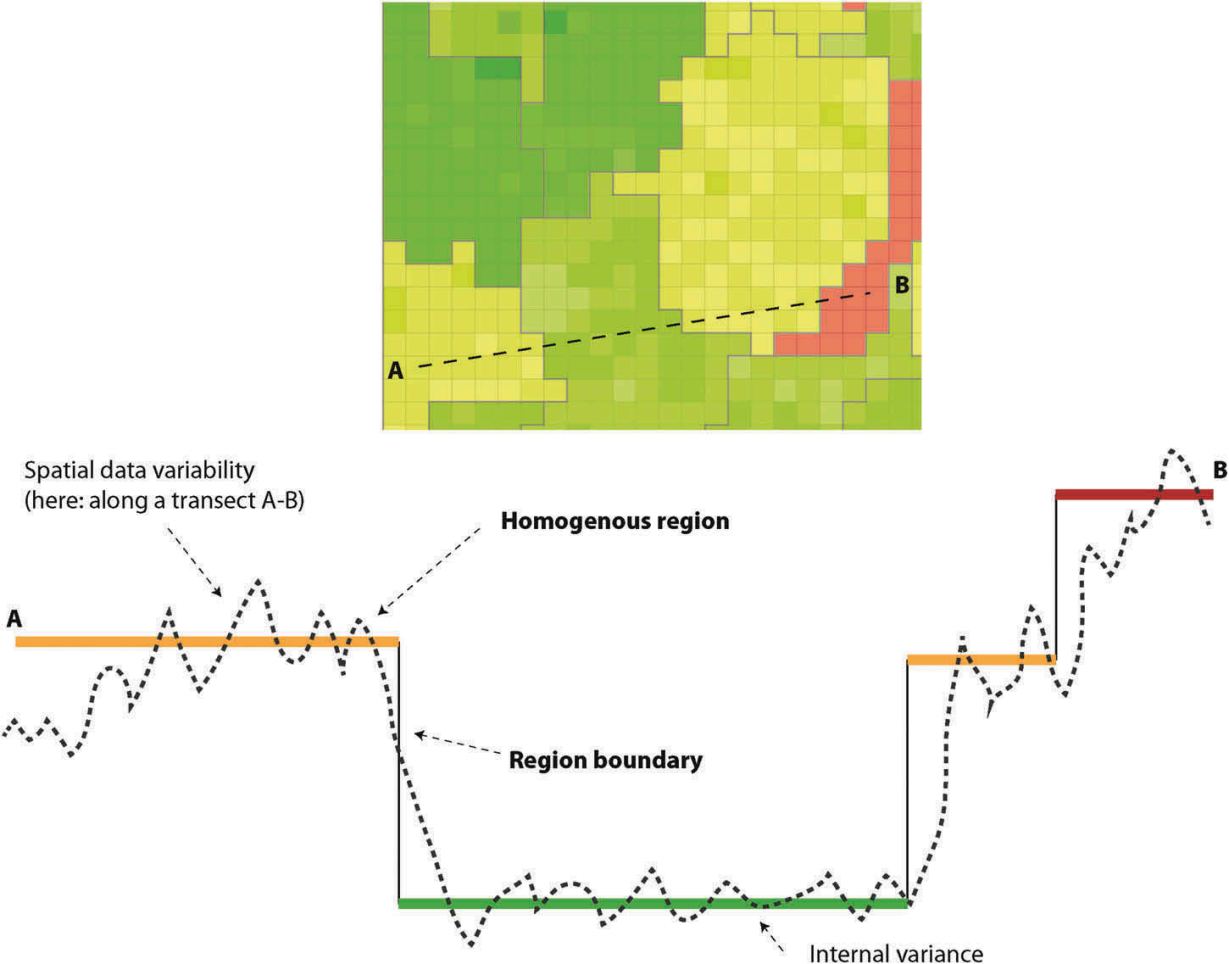
Variance-based regionalization along a transect of a spatially varying variable. Note that lower internal variance may produce larger regions.

Regionalization is a strategy for dealing with multivariate data in quantitative geography (Berry, 1967). The technique introduces boundaries along discontinuities (gradients) of spatially varying variables, grouping units (pixels, grid cells, administrative units, etc.) into regions according to a homogeneity criterion, while at the same time minimizing variance within that region. It is thereby a means to generalize multivariate spatial data from both a semantic and cartographic point of view – metaphorically carving steps in a rugged terrain (see Figure 4). Statistically, regionalization (Abler, Adams, & Gould, 1971) is a spatially constrained (Guo, 2008) stratification technique; and from a practical point of view, it is confined by the policy scale, namely a certain areal extent and respective degree of granularity (Wu & Li, 2006). The policy scale sets the scale and outer limits of the analysis; it should be considered as a constraint but not an explanatory variable for internal variability.

As outlined in the introduction, we define regions in a policy-relevant scale and scope as geons (Lang et al., 2014). The value of a geon can be an index (one-dimensional scalar) formed by combing multiple indicators (Kienberger et al., 2009), or, as in this case, EO data and non-EO data. Also, categorical values (*labels*) can be derived for the geons when discretising the underlying data to a categorical scale. Here, geons representing green valuation were derived by variance-based regionalization (see Equation 2) based on *GI_w_* assigned to 50 × 50 m grid cells. The approach builds on the multi-resolution segmentation algorithm (Baatz & Schäpe, 2000), as implemented in the software eCognition by Trimble Geospatial. Multi-scale segmentation is a key methodological element within geographic object-based image analysis (GE-)OBIA (Blaschke et al., 2014). The multi-resolution segmentation algorithm minimizes local variance (LV) within a region to be generated.

(2) $$LV_{r}=\frac{\overset{n}{\underset{c=1}{\sum }}(x_{c}-\bar{x_{c})}}{n_{r}-1}$$

where *LV_r_* equals the LV of region *r, n_R_* is the number of cells within that region, *x_c_* is the value of cell *c*, and *x_c_* represents the mean value of all cells within *r*.

In the software eCognition, the so-called scale parameter controls the maximum allowed LV within the generated regions. A method to determine the rate of change in the variance of the generated regions is the ESP tool (Drăgut, Csillik, Eisank, & Tiede, 2014), which is widely used to pre-define segmentation levels in GEOBIA. In addition, the multi-resolution segmentation uses form constraints based on the shape index (see Equation 4). After generation, the geons, whose outlines resemble the outer pixel boundaries, were straightened using the polynomial approximation with exponential kernel (PAEK) smoothing algorithm in ESRI’s ArcGIS.

In performing multi-resolution segmentation, the variance can be combined with spatial autocorrelation (SPAC, see Equation 3), whereby LV is used to characterize intra-segment homogeneity, and SPAC represents the inter-segment disparity (Kim, Madden, & Warner, 2008). A general lowering of SPAC between regions is desirable to stabilize the boundary effect. Other than in a pixel-scape, where SPAC is calculated either in a 4- or 8-neighborhood, the geon-scape consists of polygons with a variable number of adjacent neighbors. SPAC can be determined for direct neighbors or neighboring regions within a certain distance. SPAC can be measured by the spatial association statistics Global Moran’s *I*, which is defined as follows:

(3) $$I=\frac{N}{\underset{i}{\sum }\underset{j}{\sum }w_{ij}}\cdot \frac{\underset{i}{\sum }\underset{j}{\sum }w_{ij}\;\left(x_{i}-\;\overset{ˉ}{x}\right)\left(x_{j}-\;\overset{ˉ}{x}\right)}{\underset{i}{\sum }{\left(x_{i}-x\right)}^{2}}$$

where *I* is Global Moran’s I, *N* is the number of regions, *w_ij_* equals the spatial weight of the combination *i,j, x_i, j_* are the attribute values of the *i*th, *j*th region with average $\overset{ˉ}{x}$.

Here, the overall spatial association pattern of the generated geonscape was assessed by calculating Global Moran’s *I* in ArcGIS with incremental search distances. Applying 30 distance bands, the analysis was done in different distance increments, 100 m and 1000 m (see Figure 9). This incremental SPAC investigates whether the similarity of geon values is greater at certain distances than at others.

**Figure 5.**
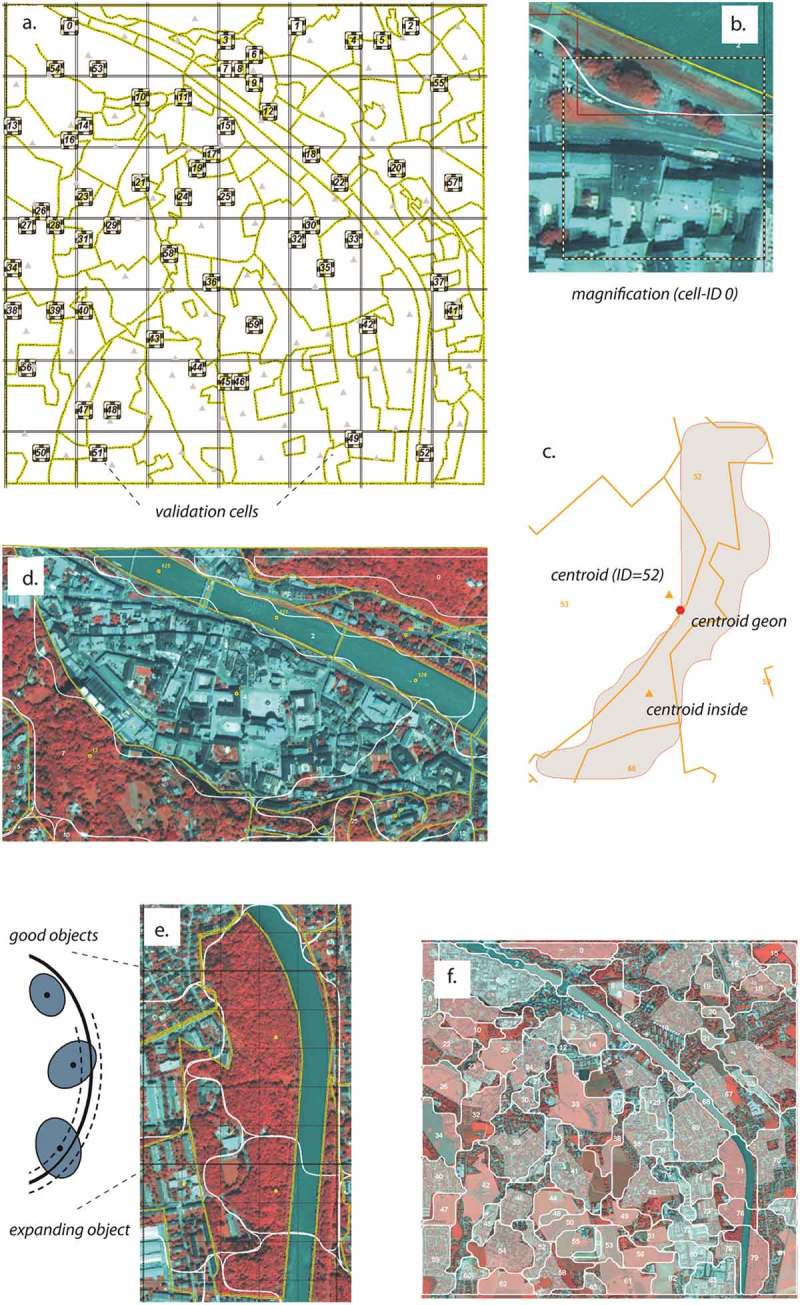
Methodological steps for validation. (a) validation grid with 60 randomly selected validation cells; (b) zoom in to cell-ID = 0 with boundaries in the upper part (reference unit in yellow, geon boundary in red, smoothed boundary in white; (c) centroid analysis, comparison of centroids of geon and reference units (including inside centroid); (d) corresponding units (yellow lines) and geons (white); (e) so-called good objects and one expanding object within one reference unit according to OFA terminology; (f) intersection of geons and reference units with largest subunit highlighted with shade. See text for further explanation.

**Figure 6.**
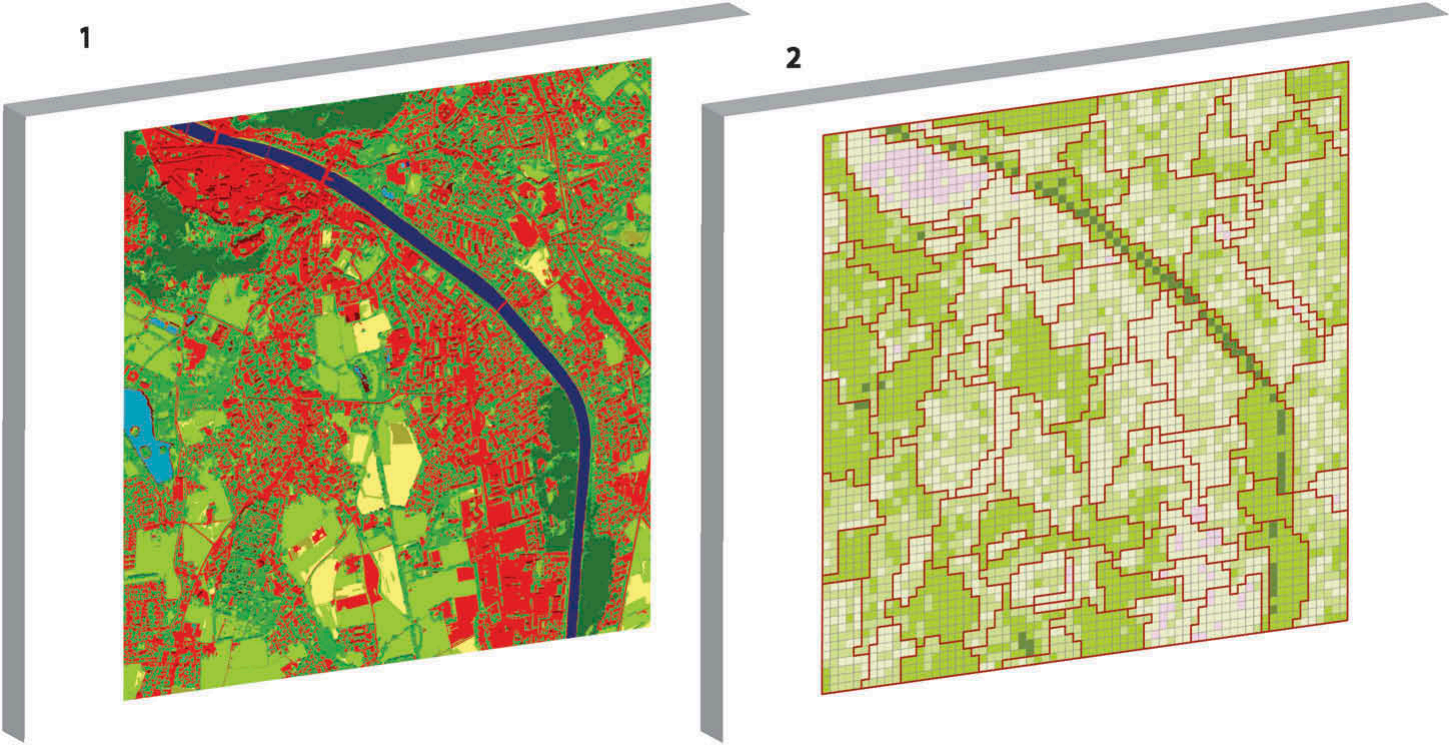
From object-based classification to a geon set representing urban green valuation.

**Figure 7.**
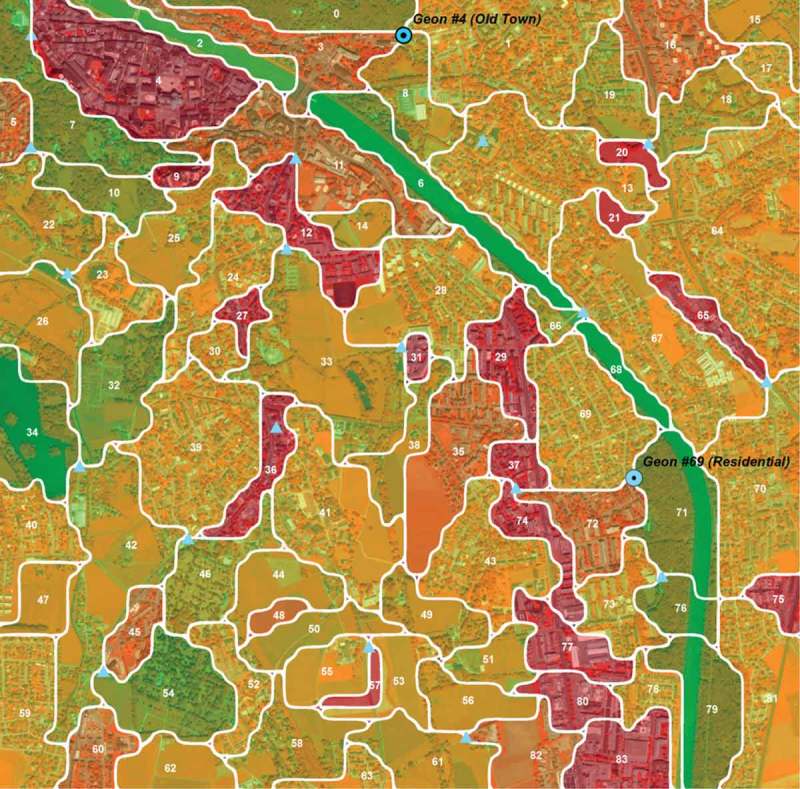
Overview of geon delineation in SBG_QB. Numbers represent the IDs of the generated geons. Small triangles indicate the places where ground validation has been performed. Circles indicate geons locations of photo-documentation (see Figure 10).

**Figure 8.**
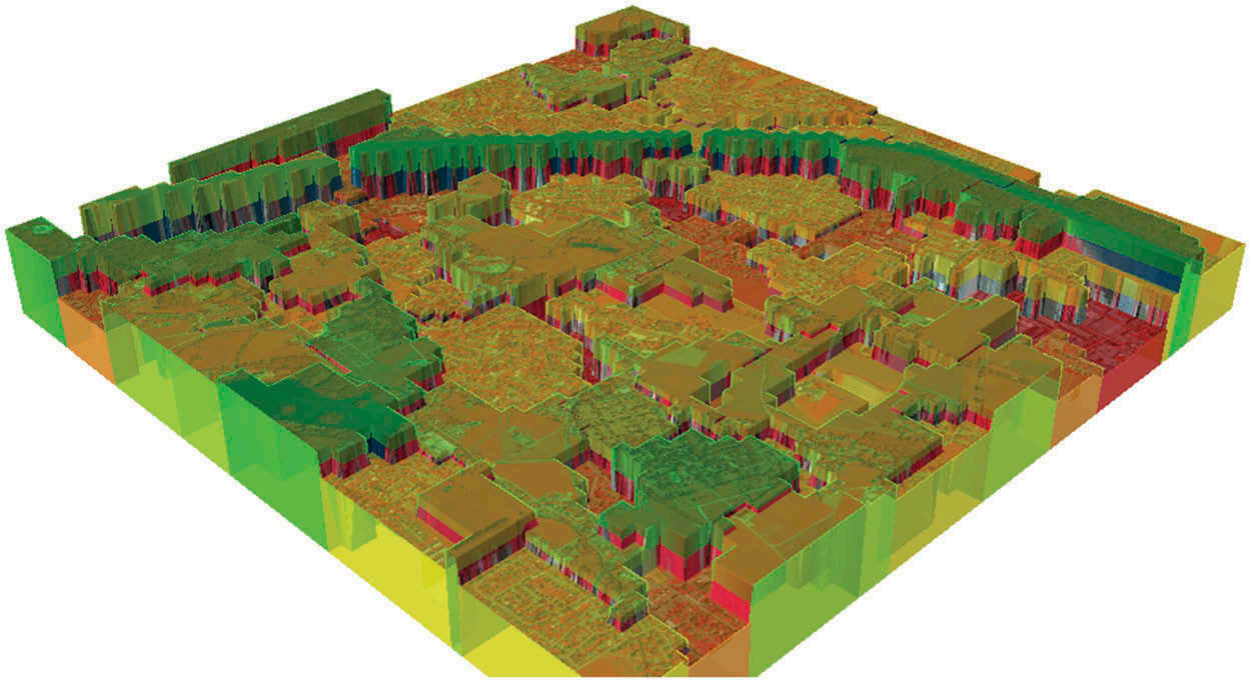
Visualization of geons, elevated according to their valuation draped over QuickBird imagery in FCIR band combination. The wooded city hills clearly stand out together with the river basin, including the riparian woodland, the ponds, and other recreational areas.

**Figure 9.**
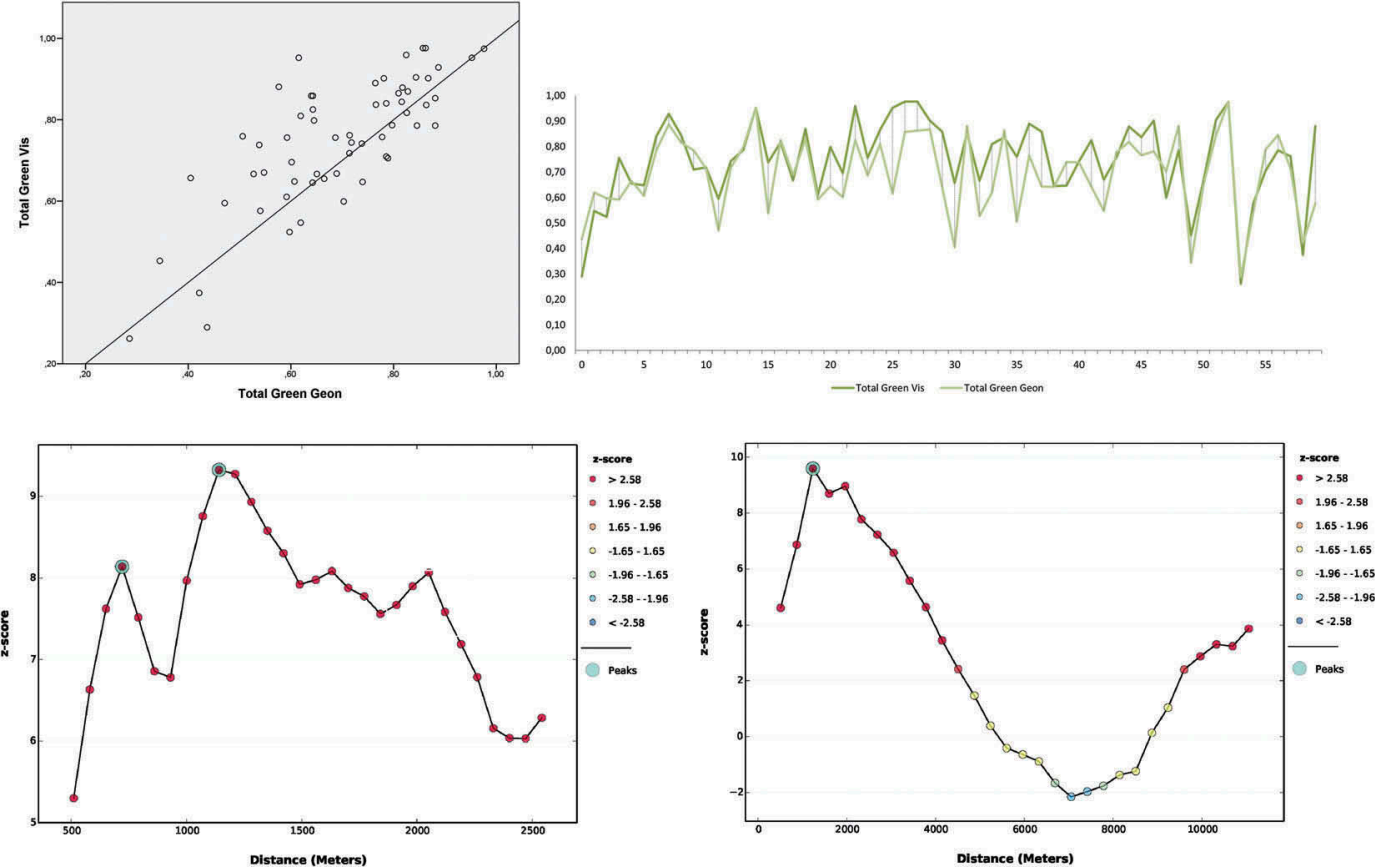
Top: scatterplot with regression line of total green (green potential) within 60 sampling grid cells. The graph on the right shows a scatterplot and deviations of valuation values for each unit. Bottom: SPAC analysis in two distance increments, 100 m (left) and 1000 m (right), with 30 incremental steps each.

Validation was done in ArcGIS 10.3.1. along the steps explained below (see Figure 5 for an overview). To serve as reference geometry, visual interpretation has been conducted by an independent expert in August 2015. The expert was trained on the visual cues indicative to certain green structures and to work in a similar target scale and applying the same principles of (green) structure homogeneity as were used for the delineation of geons. Values were estimated in the same range and measurement scale as the geons applying a scale of 1 digit (e.g. 2.8, 3.4, etc.). Four steps were performed: (1) Basic statistical descriptors such as mean and standard deviation in size and shape, range of valuation values, and variety (of classes) were calculated for both the geons and the reference units to broadly compare each type of representation. (2) A sampling grid was generated (Strasser & Lang, 2015) with regular cells, each 100 × 100 m in size. Out of the 1225 grid cells, 65 were randomly selected assuming a 90% confidence interval and a 10% sampling error. To avoid boundary effects (partial coverage at the edges of the study area), five of them were dismissed. The remaining 60 cells were retained for further analyses (see Figure 5(a,b)). The maximum possible value for green valuation has been determined as a result of the product of the cell area and the highest green valuation value (4.2), i.e. 42,000. The deviation from this maximum value is considered the actual *green potential* and expressed as percentage for each cell. IBM’s SPSS Statistics 24 was used to calculate bivariate correlation between the green potentials of the geon set and the reference units, testing the null hypothesis that there is no relationship among the calculated and the visually assessed green potentials. (3) A point-in-polygon analysis (Tiede, 2014) was carried out calculating the (inside) centroids for each of the digitized reference units and linked to the corresponding geons by perform a spatial join (Figure 5(c,d)). Value association of the centroids and the geons was again assessed by calculating a correlation coefficient. (4) Finally, geometries of the digitized reference units and the delineated geons were analyzed by a method for object-based accuracy assessment, called object-fate analysis (OFA) (Schöpfer, Lang, & Albrecht, 2008). OFA investigates the relationships of spatially corresponding reference and delineation units (Hernando, Tiede, Albrecht, & Lang, 2012), by applying a virtual overlay and then determining spatial relationship types with respect to a dynamically generated buffer around each unit (Figure 5(e)). According to the terminology proposed by Schöpfer et al. (2008), the overlapping units are spatially corresponding and called *good objects* or *expanding objects*, depending on the degree of overlap. Here we focus on *good objects* only, while two cases are differentiated, namely when the corresponding units are more or less identical (symmetric agreement), or one unit contains two or more sub-units (asymmetric agreement). In order to determine not just the number of corresponding units, but also the degree of mutual overlap, an intersection operation of the set of geon polygons *G* and set of reference units *R* was performed, resulting in a set of subunits *S* (Figure 5(f) and Equation (4)).

(4) $$SI_{GR}=G\;\cap R$$

where *SI_GR_* is the spatial intersection of *G* and *R*.

For each geon or reference unit *U*_i_, only the largest intersection was retained in a selection set *S_G/R_* (Equation 5)

(5) $$S_{G/R}=\left\{\underset{U_{1\ldots i}}{\max}\left(u_{1}\ldots u_{n}\right)\right\}$$

where *S* is the set of largest subunits within *G* or *R*.

Then the area percentages were calculated each elements of *S_G_* shares with *R*, and each element of *S_R_* shares with *G*, respectively. Based on this, the mutual agreement between geons and reference units was determined by setting a threshold for a minimum area percentage applying to both geometries (e.g. 80%).

**Figure 10.**
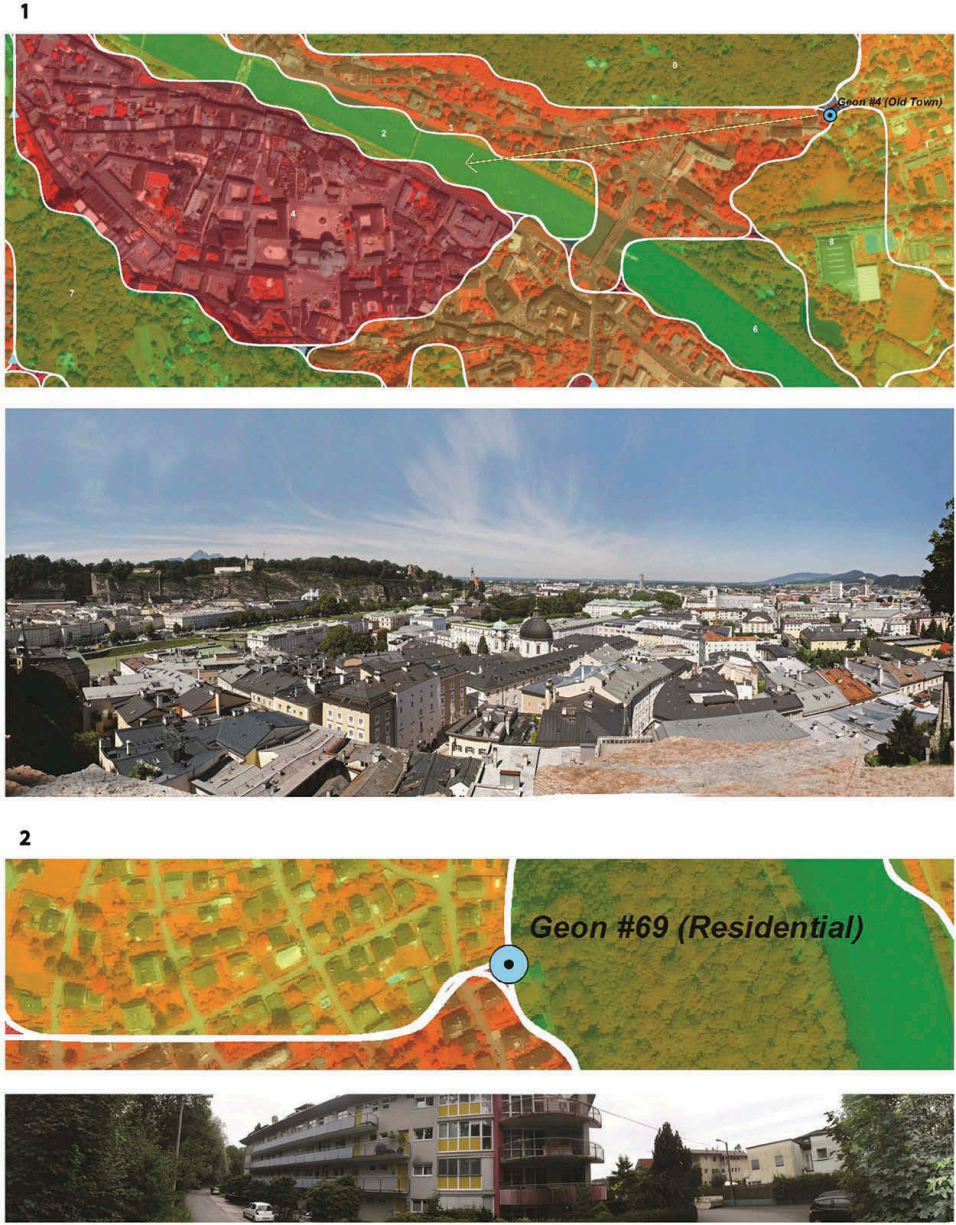
Photo documentation. Top: view from validation point on geon #4 (old town). Below: intersection of geon #69, #71, and #72 on 360° panorama photo (photographs: A. Binn).

Finally, a field campaign was carried out to assess the geon delineation, and in particular the meaningfulness of the boundaries, on the ground. A sequence of locations, preferably located at the junction or transition zone between neighboring geons, was visited and documented by 360° panorama photographs in July 2015 (see Figure 10). The dominance of the relevant green structures was assessed for plausibility.

### Spatial characteristics of geons

The arrangement of the delineated geons presents an aggregated view of the spatial distribution of the phenomenon under concern, herein equally valuated, green units. The generated set of geons (geonscape) represents a conversion from spectral reflectance into nominal classes and further on into a qualitatively ranked scalar in a range of [1|5]. The effect of the regionalization lies in both a spatial and thematic aggregation, so to stabilize the spatial variation by neglecting high frequencies while at the same time emphasizing the larger variations. The delineated geons can be re-categorized by analyzing their internal composition and accordingly deriving descriptive names; for example, geons in a residential area with a reasonable share of forest and meadows. The following metrics were selected to characterize the spatial behavior of geons: shape index and Shannon’s diversity. The shape index (Bosch, 1978) is a spatially explicit measure that directly measures the geometric shape of each individual geon. Other than a simple area-perimeter ratio, it is a standardized form metric irrespective of the actual size of a geon. Shannon’s diversity is a spatially implicit measure, characterizing each geon by its internal composition, i.e. the number and distribution of its components. The metric was chosen due to scale invariance with respect to the average size of the geons and their components.

The shape index (Equation 6) is used as a form measure, characterizing geons according to the deviation of an equally-sized circle.

(6) $$SI_{g}=\;\frac{L_{g}}{2\sqrt{\mu }A_{g}}$$

where *SI_g_* is the shape index for geon *g, A_g_* is the area, and *L_g_* is the boundary length (perimeter) of *g*.

Shannon’s diversity (Equation 7) was calculated to determine internal heterogeneity on the level of each individual geon.

(7) $$H_{g}=-\overset{n}{\underset{c=0}{\sum }}P_{c}\cdot \ln\left(P_{c}\right)$$

where *H_g_* connotes the diversity within a geon *g, P* is the percentage of each class *c, n* is the number of classes occurring within *g*.

## Results

The initial geon delineation was carried out in the smaller AOI, SBG_QB, where we used QuickBird imagery from June 2005. In the following section, the results are visualized and explained. Then, results of the four validation steps, as well as the spatial association analysis, are discussed, and examples of the qualitative ground assessment are shown. Lastly, the outcome of the transfer to the entire study area, SBG_WV based on WorldView-2, is described and analyzed.

### Geon delineation and visualization

The Figures 6–8 illustrate the process of geon delineation for SBG_QB from classification, over green index calculation, to geon generation and 3D visualization.

Figure 7 shows the geonscape for SBG_QB. The geon values represent the mean value of the cell values belonging to it; the values are classified into five classes using the natural breaks methods. The classes were assigned gradual labels (*no green, little green, fairly green, green, very green*), with graduated colors from red to green. Values ranged between 1.1 (lowest green valuation) and 4.2 (highest green valuation). The number of geons shown in this figure is 84, with an average area of 13.1 ha, ranging between 1.9 and 5.0 ha in size.

Figure 8 shows a 3D visualization of geons according to their value, i.e. their valuation. The wooded city hills clearly stand out together with the river basin, including the riparian woodland, the ponds, and other recreational areas.

### Quantitative and qualitative validation, spatial association

Table 1 shows basic statistical descriptors for a comparative analysis of the two delineation types, geons and visually interpreted reference units, and their respective composition. The number of units is about one third less for geons (84) as compared to the reference units (127), resulting in a larger average size of each unit (13.11 ha vs. 8.9 ha). Consequently, the range values of the unit sizes indicate a smaller minimum size than for geons, with altogether 16 reference units rendered smaller than the smallest geon. The average shape index is quite similar for both delineation types (1.52 vs. 1.49), while for the non-smoothened geons, due to the stepped outlines, the shape index is some 10% higher (1.72). The mean variance for LV are slightly lower for the geons (0.41 vs. 0.49), while it needs to be emphasized that geons are delineated based on an optimization of this variance (via the scale parameter), and for visual interpretation the variance is used as an indirect cue. LV was derived by zonal statistics over the weighted green index grid. The ranges of LV are similar for both delineation types. Finally, the variety of green structure types (classes) revealed the same ranges (1…6), but with a remarkably different distribution of these ranges. The distribution of classes within the geons was approximately symmetric (skewness: 0.16), while the distribution within the reference units was moderately to highly skewed toward less classes (skewness: 0.96)

**Table 1.** Basic statistical descriptors for the units of the two delineation types.

Delineation type	# of units	Mean size (ha)	Range size (ha)	Mean *SI*	Mean *LV*	Range *LV*	Range *CV*	Skewness *CV*
Geons	84	13.11 (13.16)	[19.3 | 49.9]	1.52 (1.72)	0.41	[0.28 | 3.11]	[1| 6]	0.16
Visual interpretation	127	8.9	[4.4 | 44.3]	1.49	0.49	[0.36 | 3.14]	[1|6]	0.96

SI: shape index; LV: local variance; CV: class variety; ha: hectares. In parentheses: results for the non-smoothed geons. Number in brackets indicated ranges.

The results of the sampling grid analysis, which compares the green potentials of both delineations, are shown in Figure 9. A scatterplot including a regression line was produced as well as a chart showing the deviations of green potential per unit. Between the two delineation types, the overall agreement (Pearson product-moment) was calculated as 0.77 (*n* = 60), significant at the 0.01 level.

As a third step of the validation, the centroid (point-in-polygon) analysis revealed a correlation coefficient of 0.69 (*n* = 127), showing high agreements of the corresponding geometries. The last part of quantitative validation was the OFA assessment, using a dynamic buffer size of 10, meaning that the buffer distance is calculated dynamically in dependence of the polygon area, in this case 10% of the area. Out of 581 intersecting units, the selection set *S*_G_ holds the 84 units which show the largest intersection areas. Those areas were further analyzed in terms of their mutual agreement between the two delineation types. Out of these 84 units, 58 show an agreement level of >0.5, i.e. at least 50% area percentage on each corresponding units. Altogether, 45 show a good match with more than 60% area overlap, from which 10 show a high match with more than 80%. Another 24 have an asymmetric agreement with a minimum area percentage of 80% on the larger unit and a maximum area percentage of 50% on the smaller unit (see Table 2 for details).

**Table 2.** OFA assessment in SBG_QB (total number of geons = 84).

Relation type	Agreement type	Agreement level	No. of cases	Example
1:1 (Geons ~ visual)	Symmetric	0.8 (High match)	10	
		0.6 (Good match)	35	
1:n (Geons < visual)	Asymmetric	> 0.8 AND < 0.5	13	
n:1 (Visual > geons)	Asymmetric	< 0.5 AND > 0.8	11

**Table 3.** Selected metrics for the entire geon set (SBG_WV), and individual geons (total number of geons = 1083).

Min	Max	Mean	Std-dev	Kurtosis (histogram)
*Geon value (green valuation)*				
0.63	4.22	2.84	0.78	−0.28 (bimodal)
*Area statistics (hectares)*				
0.49	66.49	13.54	9.41	1.41 (unimodal)
*Form (shape index)*				
1.10	4.05	1.57	0.28	1.70 (unimodal)
*Diversity (including all classes)*				
0	2.11	1.24	0.47	−0.63 (unimodal)

Figure 9 shows the results of the incremental SPAC analysis to identify spatial association patterns. The two graphs indicate a first peak can be detected at around 700 m and a second peak around 1200 m, even more pronounced, at around 1100 m. On a larger scale, SPAC decreases after these peaks until a distance range of around 7000 m, before it increases again.

The ground check performed by photo documentation took place at 20 locations distributed randomly at intersection points of the geon boundaries (see Figure 8 above, blue triangles, not all shown). 360° photo stitching was performed, and short interviews were conducted with locals, asking about some general living conditions (perceived degree of greenery and building density, soundscape, etc.). Figure 10 shows an example of this documentation.

### Expansion to full study area and geonalytics

**Figure 11.**
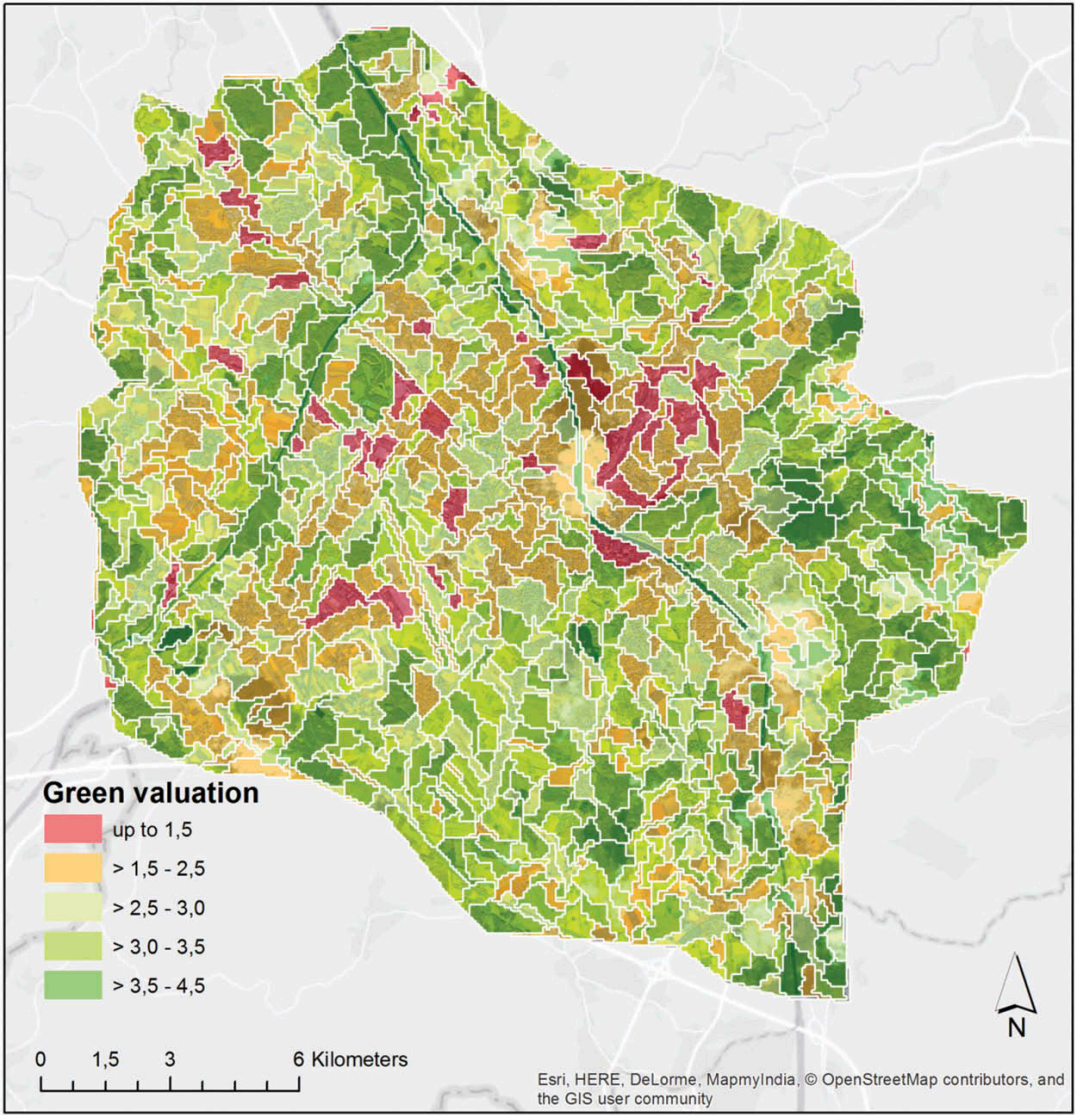
Geonscape for the masterplan region SBG_WV.

For further analysis, the geon delineation designed for SBG_QB was transferred to the larger area, SBG_WV, covered by a WorldView-2 scene (Figure 11). The edge effect arising due to the irregular study area was taken into account, and borderline polygons were removed by inverse buffering.

Table 3 shows the geonalytics results on geon level. It contains average values, ranges for the descriptors value distribution, size (area in hectares), shape index, and Shannon’s diversity. Note that the histogram of the geon values in a range from [1|5] shows a bimodal distribution, with local maxima at around 2.4 and 3.5. Note also that the maximum H value is *ln* (13) = 2.56, as in the natural logarithm of the maximum number of classes occurring in the area.

**Figure 12.**
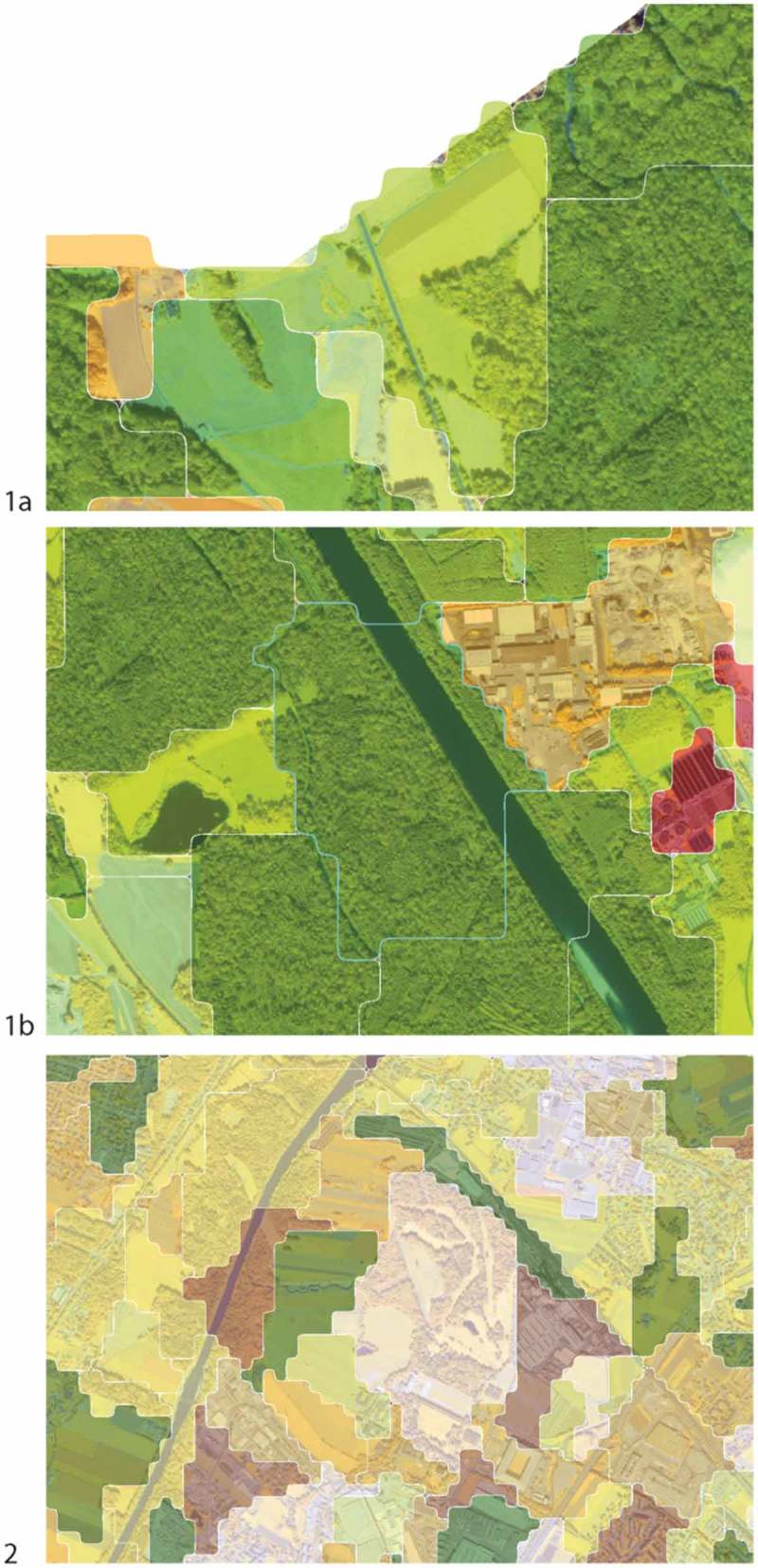
Internal structure and derived diversity values and categorization. (1a + b) geons categorized as forest edge (co-occurrence of meadow and forest) and riparian forest (co-occurence of forest river); (2) graduated color visualization of Shannon’s diversity (grey/low – yellow – green/high).

Figure 12 shows a selection of geons and their internal structure resulting in different Shannon’s diversity values and correspondingly to a specific categorization.

## Discussion and outlook

This section highlights the potential of the approach before discussing some limitations and critical steps in the work performed, along with some suggestions to improve workflows in the future, in particular when moving on to the upcoming time-step to be based on 2015 Pléiades stereo data. The scale parameter, controlling for a maximum variance at the geon generation, limits the variation of unit areas, so that altogether, a more appropriate scaled representation can be achieved. In some areas, the visual interpretation tends to introduce smaller units, as compared to the geons, and thus the heterogeneity of neighborhoods may be (over-)emphasized. Also, it became obvious that visual interpretation intuitively tends to respect boundaries (roads, rivers), while the geon delineation transgresses these. Even if roads (in particular larger ones) do have some barrier effects, left and right of those features the situation is often comparable, e.g. when the road has dissected a former functional unit. Other linear features like the river Salzach may have an integrative effect anyway, with respect to green valuation.

The approach relies on several steps, with user-defined parameters and specifications. Several limitations are implied. At first, the set of classes was case-defined in this study. For a better comparability and transferability to similar urban settings (e.g. it is planned to use a similar approach in the similar sized city of Szeged in Hungary), it is recommended to use a more standardized classification set, preferably building on an existing and established scheme such as the Urban Atlas categories, and then adding more detailed levels. Second, proper image pre-processing including image calibration is critical for the results not to be biased by spectral peculiarities. It needs to be noted that the input data (QuickBird and WorldView-2) for 2005 and 2010 were only partly calibrated and atmospherically corrected; complete pre-processing has been done for the Pléiades data for 2015. Third, the scale of the geons’ allowed internal variance will influence the outcome; here, a better empirical basis is provided by the incremental SPAC analysis. The principal idea of regionalization is that existing boundaries, predominantly administrative ones on the community, provincial, or state level, would not reveal the true spatial character of the underlying phenomenon, but rather obscure and bias its actual distribution. Instead of further fuelling the *modifiable areal unit problem* (MAUP) (Openshaw, 1984), the geon concept was designed to overcome it, by representing the phenomenon’s actual spatial variation (Lang et al., 2014). In this study, SPAC was used as an analytical tool, not for delineation. Regionalization necessarily leads to low autocorrelation among neighboring regions through the boundary effect. So SPAC may be used in the future for the delineation itself. Fourth, the intermediate step of referring the weighted green index to regular grid cells could be optimized. Rather than using regular grid cells, methods of superpixel generation, such as SLIC (Achanta et al., 2012) could be used to create an intermediate level that is closer to the actual spectral characteristics of the underlying image. The seed pixels could match the centroid of (multiples of) the European reference grid as proposed by Lang and Csillik (2017). Sixth, the construction of geons differs from classic spatial predictive modeling as not the occurrence of a specific, measurable variable is predicted over space, based on point observations and interpolation, but a latent phenomenon is mapped by integrating various layers of representation. Thereby, the generated geons can hardly be directly validated in binary mode as right or wrong, but rather as appropriate, suitable, or meaningful. Plus, an evaluation of the delineated units is more feasible the lower the data scale is. A pure quantitative value of an average green valuation is difficult to assess on the ground, while a gradual legend (not green, little green, etc.) is easier to assess against ground reference. Even more is a categorical label (e.g. riparian forest dominated). When comparing the automated delineation with a visual interpretation, geometric overlay assessment can provide a quantitative measure of the agreement in a spatially explicit way. In the future, it is foreseen to expand the study to further criteria of urban valuation, including aspects, such as aesthetics of the urban structures. Most probably, when considering aesthetic, a higher value may be assigned to geons which, characterized by distinct architectural features, even bearing a low percentage of greenery.

## Conclusions

In this paper a strategy was discussed to represent areas of uniform green valuation under certain size and homogeneity constraints. The outcome of this method based on the geon approach is a map of urban green valuation that reveals qualitative aspects of urban green as a latent spatial phenomenon. Integrating qualitative assessments with remote sensing data in a geon-scape, the generated units are homogeneous building blocks for urban planning purposes, exhibiting place-related characteristics, as well as the overall urban-scale variability of the concerned phenomenon.

## Geolocation information

The centroid of the study area is located at 47.8 N/13.0372 E (decimal degrees, WGS).

## References

[CIT0001] Abler R., Adams J.S., & Gould P. (1971). *Spatial organization - the geographer’s view of the world*. Englewood Cliffs: Prentice-Hall.

[CIT0002] Achanta R., Shaji A., Smith K., Lucchi A., Fua P., & Süsstrunk S. (2012). SLIC superpixels compared to state-of-the-art superpixel methods. *IEEE Transactions on Pattern Analysis and Machine Intelligence*, 34, 2274–2282. doi:10.1109/TPAMI.2012.120 22641706

[CIT0003] Allen T.F.H., & Starr T.B. (1982). *Hierarchy*. Chicago: University of Chicago Press.

[CIT0004] Baatz M., & Schäpe A. (2000). *Multiresolution segmentation: An optimization approach for high quality multi-scale image segmentation*. Salzburg: Wichmann Verlag.

[CIT0005] Berry B.J.L. (1967). Grouping and regionalizing: An approach to the problem using multivariate analysis In Garrison W.L. & Marble D.F. (Eds.), *Quantitative geography* (pp. 219–251). Evanston: Northwestern Studies in Geography.

[CIT0006] Blaschke T., Hay G.J., Kelly M., Lang S., Hofmann P., Addink E., ... Tiede, D (2014). Geographic object-based image analysis: A new paradigm in remote sensing and geographic information science. *International Journal of Photogrammetry and Remote Sensing*, 87(1), 180–191. doi:10.1016/j.isprsjprs.2013.09.014 PMC3945831 24623958

[CIT0007] Bosch W. (1978). A procedure for quantifying certain geomorphological features. *Geographical Analysis*, 10, 241–247. doi:10.1111/j.1538-4632.1978.tb00653.x

[CIT0008] Byrne D. (1998). *Complexity theory and the social sciences - an introduction*. London: Routledge.

[CIT0009] Carrus G., Scopelliti M., Lafortezza R., Colangelo G., Ferrini F., Salbitano F., ... Sanesi, G. (2015). Go greener, feel better? The positive effects of biodiversity on the well-being of individuals visiting urban and peri-urban green areas. *Landscape and Urban Planning*, 134, 221–228.

[CIT0010] Drăgut L., Csillik O., Eisank C., & Tiede D. (2014). Automated parameterisation for multi-scale image segmentation on multiple layers. *ISPRS International Journal of Photogrammetry and Remote Sensing*, 88, 119–127. doi:10.1016/j.isprsjprs.2013.11.018 PMC3990455 24748723

[CIT0011] Grimm N.B., Faeth S.H., Golubiewski N.E., Redman C.L., Wu J., Bai X., & Briggs, J. M. (2008). Global change and the ecology of cities. *Science*, 319(5864), 756–760. doi:10.1126/science.1150195 18258902

[CIT0012] Guo D. (2008). Regionalization with dynamically constrained agglomerative clustering and partitioning (REDCAP). *International Journal of Geographical Information Science*, 22, 801–823. doi:10.1080/13658810701674970

[CIT0013] Hagenlocher M., Kienberger S., Lang S., & Blaschke T. (2014). Implications of spatial scales and reporting units for the spatial modelling of vulnerability to vector-borne diseases In Vogler R., Car A., Strobl J., & Griesebner G. (Eds.), *GI_Forum 2014. Geospatial Innovation for Society* (pp. 197–206). BerlIn Wichmann Verlag.

[CIT0014] Hecht R., Meinel G., & Buchroithner M. (2008). Estimation of urban green volume based on single-pulse lidar data. *IEEE Transactions on Geoscience and Remote Sensing,* 46(11), 3832–3840.

[CIT0015] Hernando A., Tiede D., Albrecht F., & Lang S. (2012). Spatial and thematic assessment of object-based forest stand delineation using an OFA-matrix. *International Journal of Applied Earth Observation and Geoinformation*, 19, 214–225. doi:10.1016/j.jag.2012.05.007

[CIT0016] Kaplan S. (1995). The restorative benefit of nature: Toward an integrative framework. *Journal of Environmental Psychology*, 15, 169–182. doi:10.1016/0272-4944(95)90001-2

[CIT0017] Keul A., & Prinz T. (2011). The Salzburg quality of urban life study with GIS support In Marans R.W. & Stimson R.J. (Eds.), *Investigating quality of Urban life – Theory, methods, and empirical research* (pp. 273–293). Dordrecht: Springer.

[CIT0018] Kienberger S., Lang S., & Zeil P. (2009). Spatial vulnerability units – Expert-based spatial modeling of socio-economic vulnerability in the Salzach catchment, Austria. *Natural Hazards and Earth System Sciences*, 9, 767–778. doi:10.5194/nhess-9-767-2009

[CIT0019] Kim M., Madden M., & Warner T.A. (2008). Estimation of optimal image object size for the segmentation of forest stands with multispectral IKONOS imagery In Blaschke T., Lang S., & Hay G. (Eds.), *Object-based image analysis - Spatial concepts for knowledge-driven remote sensing applications* (pp. 291–307). BerlIn Springer.

[CIT0020] Koestler A. (1967). *The ghost in the machine*. London: Hutchinson.

[CIT0021] Kuhn W., & Ballatore A. (2015). Designing a language for spatial computing In Bação F., Santos M.Y., & Painho M. (Eds.), *AGILE 2015 - Geographic Information Science as an enabler of smarter cities and communities* (pp. 309-326)). Berlin: Springer International.

[CIT0022] Lang S., & Csillik O. (2017). ETRF grid-constrained superpixels generation in urban areas using multi-sensor very high resolution imagery *GI Forum* . *Journal for Geographic Information Science*, 2/2017 244–252.

[CIT0023] Lang S., Jekel T., Hölbling D., Schöpfer E., Prinz T., Kloyber E. & Blaschke T. (2006). *Where the grass is greener–Mapping of urban green structures according to relative importance in the eyes of the citizens* In: P. Hostert, S. Schiefer & A. Damm (Eds.), *Workshop of the EARSeL special interest group on Urban remote sensing,“Challenges and Solutions”*. CD-ROM, Berlin

[CIT0024] Lang S., Kienberger S., Tiede D., Hagenlocher M., & Pernkopf L. (2014). Geons – Domain-specific regionalization of space. *Cartography and Geographic Information Science*, 41(3), 214–226. doi:10.1080/15230406.2014.902755

[CIT0025] Lang S., Schöpfer E., Hölbling D., Blaschke T., Moeller M., & Jekel T. (2007). Quantifying and qualifying urban green by integrating remote sensing, GIS, and social science methods In Petrosillo I., Müller F., Jones K.B., Zurlini G., Krauze K., Victorov S., … Kepner L. Bai-Lian & W.G., et al, (Eds.). *Use of landscape Sciences for the assessment of environmental security* (pp. 93–105). Springer.

[CIT0026] Luederitz C., Lang D.J., & Von Wehrden H. (2013). A systematic review of guiding principles for sustainable urban neighborhood development. *Landscape and Urban Planning*, 113, 40–52. doi:10.1016/j.landurbplan.2013.06.002

[CIT0027] Nielsen M.M. (2015). Remote sensing for urban planning and management: The use of window-independent context segmentation to extract urban features in Stockholm. *Computers, Environment and Urban System*, 52, 1–9. doi:10.1016/j.compenvurbsys.2015.02.002

[CIT0028] Nir D. (1987). Regional geography considered from the system’s approach. *Geoforum*, 18(2), 187–202. doi:10.1016/0016-7185(87)90005-4

[CIT0029] O’Neill R., DeAngelis D., Waide J., & Allen T.F.H. (1986). *A hierarchical concept of ecosystems*. Princeton: Princeton University Press.

[CIT0030] Openshaw S. (1984). *The modifiable areal unit problem* (Vol. 38). Norwich: Geo Books.

[CIT0031] Powell D. (2011). *Deriving an urban green index from object-based classification of very high resolution remote sensing imagery with different conditions of illumination*. Salzburg: MSc, University of Salzburg.

[CIT0032] Schöpfer E., Lang S., & Albrecht F. (2008). Object-fate analysis - spatial relationships for the assessment of object transition and correspondence In Blaschke T., Lang S., & Hay G.J. (Eds.), *Object-based image analysis - spatial concepts for knowledge-driven remote sensing applications* (pp. 785–801). BerlIn Springer.

[CIT0033] Strasser T., & Lang S. (2015). Object-based class modelling for multi-scale riparian forest habitat mapping. *International Journal of Applied Earth Observation and Geoinformation*, 37, 29–37. doi:10.1016/j.jag.2014.10.002

[CIT0034] Tiede D. (2014). A new geospatial overlay method for the analysis and visualization of spatial change patterns using object-oriented data modeling concept. *Cartography and Geographic Information Science*, 41, 227–234. doi:10.1080/15230406.2014.901900 27019643 PMC4786846

[CIT0035] Tomanev J. (2009). *Region* (pp. 136–150). Oxford: Elsevier.

[CIT0036] Weinberg G.M. (1975). *An introduction to general systems thinking*. New York: Dorset House Publishing.

[CIT0037] Wiens J. (1989). Spatial scaling in ecology. *Functional Ecology*, 3(4), 385–397. doi:10.2307/2389612

[CIT0038] Wu J., & Li H. (2006). Concepts of scale and scaling In Wu J., Jones K.B., Li H., & Loucks O.L. (Eds.), *Scaling and uncertainty analysis in ecology: Methods and applications*. BerlIn Springer.

[CIT0039] Zadeh L.A. (1965). Fuzzy sets. *Information and Control*, 8(3), 338–353. doi:10.1016/S0019-9958(65)90241-X

